# Incidence of Herpes Zoster and Postherpetic Neuralgia and Herpes Zoster Vaccination Uptake in a US Administrative Claims Database

**DOI:** 10.1093/ofid/ofae211

**Published:** 2024-04-16

**Authors:** Zachary A Marcum, Purva Jain, Alan Embry, Brent Arakaki, Irisdaly Estevez, Emma Viscidi

**Affiliations:** Science, Aetion, Inc, New York, New York, USA; Science, Aetion, Inc, New York, New York, USA; Clinical Development–Infectious Diseases, Moderna, Inc, Cambridge, Massachusetts, USA; Science, Aetion, Inc, New York, New York, USA; Science, Aetion, Inc, New York, New York, USA; Clinical Development–Infectious Diseases, Moderna, Inc, Cambridge, Massachusetts, USA

**Keywords:** herpes zoster, postherpetic neuralgia, shingles, vaccination, immuncompromised

## Abstract

**Background:**

The objective of this study was to estimate the annual incidence rates of herpes zoster (HZ) and postherpetic neuralgia (PHN) among individuals aged ≥19 years and the proportion who received HZ vaccination among those aged ≥50 years.

**Methods:**

This observational cohort study was conducted with administrative claims data from HealthVerity and included insured individuals across the US. Crude and US age- and sex-standardized incidence rates of HZ and PHN were calculated from 1 January 2019 to 31 May 2022 by calendar year in persons aged ≥19 years. Outcomes were defined as ≥1 *ICD-10* diagnosis code for HZ or PHN. Analyses were stratified by age, sex, and immunocompromised status. Among those aged ≥50 years, the proportion who received 1 or 2 doses of recombinant zoster vaccine (Shingrix) or 1 dose of Zostavax was calculated.

**Results:**

Standardized annual incidence rates from 2019 to 2021 were 542 to 685 per 100 000 person-years for HZ and 35 to 38 per 100 000 person-years for PHN. Rates were highest among females, older adults, and individuals who were immunocompromised. From 1 January 2019 to 31 May 2022, 4.3% and 9.0% of persons aged ≥50 years received 1 and 2 doses of Shingrix, respectively, and 0.2% received 1 dose of Zostavax.

**Conclusions:**

In this US claims database analysis, HZ and PHN were more frequent among older adults, females, and individuals who were immunocompromised. Between 1 January 2019 and 31 May 2022, 9% of persons aged ≥50 years received 2 doses of the Shingrix vaccine. Greater efforts are needed to increase vaccine uptake against HZ, especially for those at highest risk.

## Teaser

Herpes zoster and postherpetic neuralgia incidence decreased during the COVID-19 pandemic. From 2019 to 2021, herpes zoster and postherpetic neuralgia incidence rates were highest among females, older adults, and individuals who were immunocompromised. Current vaccination against herpes zoster is low among older adults.

## Graphical Abstract

**Graphical abstract:**
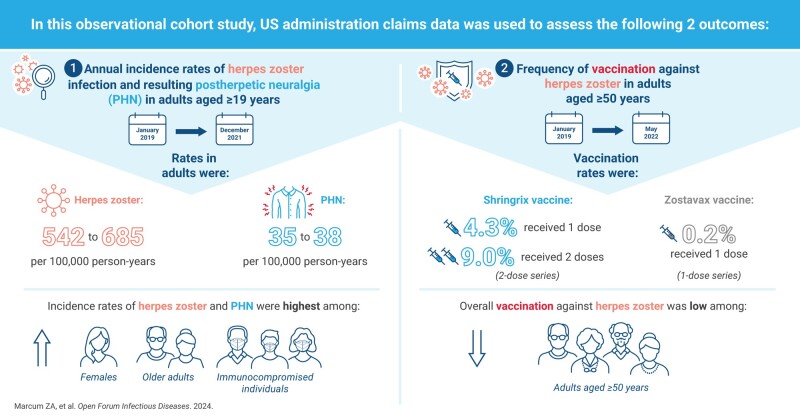

Herpes zoster (HZ), or shingles, is caused by the varicella zoster virus and occurs in an estimated 20% to 30% of adults [1, 2]. Risk of HZ increases with age, as varicella zoster virus–specific cell-mediated immunity declines [3–5]. Approximately up to 18% of individuals with HZ will develop postherpetic neuralgia (PHN), which can decrease function and quality of life [3]. HZ vaccination is currently recommended in the United States for those aged ≥50 years, as well as persons aged ≥19 years with weakened immune systems [6].

Previous studies of HZ among the general US population (ie, non–disease specific) were based on data before 2018; thus, contemporary data on HZ are limited [4, 5, 7]. Contemporary epidemiologic evidence in the United States is needed to understand the disease burden of shingles and PHN in particular due to the continued aging of the US population. This study aimed to estimate annual incidence rates of diagnosed HZ and PHN among US adults aged ≥19 years from 2019 to 2021 and to estimate uptake of the HZ vaccination from 2019 to 2022.

## METHODS

This observational cohort study was conducted with US administrative medical and pharmacy claims data from HealthVerity and included insured individuals (commercial, Medicare Advantage, or Medicaid plans). The data were limited to 2 closed claims databases, Private Source 17 and Private Source 20, with a data range from 1 December 2017 to 31 May 2022. This study was deemed exempt from full review by the WIRB-Copernicus Group institutional review board.

### Study Sample and Follow-up

The study design is depicted in Supplementary Figure 1. The study included adults aged ≥19 years to capture the range of those at greatest risk for acquiring HZ. Age and sex were assessed on the index date; individuals were excluded if their data had missing or conflicting sex or missing age. The index date within the calendar year of interest (2019, 2020, or 2021) was the day following 180 days of continuous enrollment (no gaps allowed). The baseline period included the start of all available data for a patient in the database from 1 December 2017, or later, through the index date. Outcomes were assessed over the course of the calendar year of interest, following the index date. A washout for the outcome of interest was applied prior to the index date to capture incident cases of HZ and PHN only and to exclude patients with prior HZ or PHN diagnosis. The washout period was assessed from 1 December 2017 (start of data range) onward such that the minimum washout period for any individual was 13 months.

When HZ vaccination was assessed, the entire data range (1 January 2019–31 May 2022) was used to maximize capture of vaccine exposure; thus, individuals in that analysis were required to have continuous enrollment from 1 January 2019 to 31 May 2022.

### Outcomes

HZ and PHN were separately defined as at least 1 *ICD-10* diagnosis code for HZ or PHN (any position, inpatient or outpatient setting). HZ vaccinations were defined as ≥1 dose of zoster vaccine live (Zostavax) or 1 or 2 doses of recombinant zoster vaccine (Shingrix; second dose, 30 to 210 days after the first) and were defined by *Current Procedural Terminology* codes and brand names. The measurement approach for defining HZ vaccinations is illustrated in Supplementary Figure 2. The codes utilized to measure HZ, PHN, and HZ vaccinations are included in Supplementary Table 1.

### Statistical Analyses

All analyses were conducted on Aetion Substantiate, a scientifically validated analytic platform. Baseline demographic and patient characteristics, including age, sex, and clinical conditions, were assessed. Summary statistics included mean and SD for continuous variables and counts and proportions for categorical variables. Baseline demographics and clinical characteristics are described for all individuals in 2019, 2020, and 2021.

Incidence rates of HZ and PHN were estimated separately for each calendar year and reported per 100 000 person-years. Crude estimates of incidence rates were calculated by dividing the number of patients meeting the case definition of incident HZ or PHN by the total number of person-years within the calendar year. Overall incidence rate estimates for HZ and PHN were age and sex standardized according to 2019 US Census distributions. Analyses were stratified by age, sex, and immunocompromised categories. Immunocompromised status was based on claims indicating at least 1 of the following (relative to index): blood/stem cell transplant in the prior 2 years, history of organ transplant and immunosuppressive therapy in the prior 60 days, active cancer treatment on cohort entry with an active cancer diagnosis in the prior year, any history of primary immunodeficiency, or any history of HIV infection [8].

The count and percentage of HZ vaccinations (1 dose of Zostavax, 1 dose of Shingrix, or 2 doses of Shingrix) from 1 January 2019 to 31 May 2022 were reported overall and by age categories. The entire data range was used for assessing vaccine uptake to maximize outcome ascertainment.

## RESULTS

### Incidence Rates of HZ and PHN

The HZ analytic cohorts in 2019, 2020, and 2021 included 38 471 527, 35 783 632, and 29 733 319 individuals, respectively, and the PHN analytic cohorts included 38 477 362, 35 799 297, and 29 752 654 (Table 1, Supplementary Tables 2 and 3). In 2019, the mean (SD) age was 56.0 (15.7) years for those with HZ and 63.1 (15.0) years for those with PHN. When compared with those without HZ or PHN, those with HZ or PHN were older and more likely to be female, have comorbidities, and be immunocompromised. Distributions of baseline characteristics were similar in the 2020 and 2021 cohorts.

**Table 1.** Baseline Characteristics of Patients With Incident Herpes Zoster or Postherpetic Neuralgia (2019)

	Herpes Zoster (n = 38 471 527)		Postherpetic Neuralgia (n = 38 477 362)	
	No (n = 38 283 283)	Yes (n = 188 244)	No (n = 38 467 713)	Yes (n = 9649)
Age, y, mean (SD)	46.39 (17.23)	56.04 (15.72)	46.43 (17.23)	63.17 (14.96)
Age categories, y				
19	1 259 988 (3.3)	522 (0.3)	1 260 530 (3.3)	6 (0.1)
20–24	3 302 350 (8.6)	3007 (1.6)	3 305 438 (8.6)	31 (0.3)
25–29	3 420 131 (8.9)	6590 (3.5)	3 426 793 (8.9)	108 (1.1)
30–34	3 396 931 (8.9)	9483 (5.0)	3 406 515 (8.9)	218 (2.3)
35–39	3 405 749 (8.9)	11 608 (6.2)	3 417 532 (8.9)	283 (2.9)
40–44	3 176 023 (8.3)	12 496 (6.6)	3 188 577 (8.3)	374 (3.9)
45–49	3 431 833 (9.0)	16 560 (8.8)	3 448 394 (9.0)	594 (6.2)
50–54	3 597 071 (9.4)	21 356 (11.3)	3 618 387 (9.4)	875 (9.1)
55–59	3 946 127 (10.3)	26 955 (14.3)	3 972 801 (10.3)	1311 (13.6)
60–64	3 833 251 (10.0)	28 150 (15.0)	3 860 722 (10.0)	1555 (16.1)
65–69	2 140 699 (5.6)	17 017 (9.0)	2 156 975 (5.6)	1138 (11.8)
70–74	1 367 071 (3.6)	13 127 (7.0)	1 379 351 (3.6)	1083 (11.2)
75–79	930 044 (2.4)	9561 (5.1)	938 932 (2.4)	824 (8.5)
80–84	297 959 (0.8)	3323 (1.8)	300 976 (0.8)	347 (3.6)
≥85	778 056 (2.0)	8489 (4.5)	785 790 (2.0)	902 (9.3)
Sex				
Male	17 737 533 (46.3)	68 953 (36.6)	17 805 322 (46.3)	3429 (35.5)
Female	20 545 750 (53.7)	119 291 (63.4)	20 662 391 (53.7)	6220 (64.5)
Payer type				
Commercial	23 962 084 (62.6)	114 231 (60.7)	24 076 940 (62.6)	4440 (46.0)
Medicaid	3 970 740 (10.4)	37 454 (19.9)	4 005 547 (10.4)	3228 (33.5)
Medicare	11 379 748 (29.7)	43 769 (23.3)	11 421 176 (29.7)	2541 (26.3)
Other/unknown	533 889 (1.4)	1546 (0.8)	535 427 (1.4)	50 (0.5)
Quan-Charlson Comorbidity Index score^a^				
0	31 031 689 (81.1)	120 199 (63.9)	31 151 370 (81.0)	4766 (49.4)
1	3 485 415 (9.1)	25 801 (13.7)	3 510 406 (9.1)	1547 (16.0)
≥2	3 766 179 (9.8)	42 244 (22.4)	3 805 937 (9.9)	3336 (34.6)
Comorbidities				
Alcohol use	570 588 (1.5)	3236 (1.7)	573 679 (1.5)	192 (2.0)
Arrhythmia	2 132 973 (5.6)	22 551 (12.0)	2 154 284 (5.6)	1639 (17.0)
Asthma	1 778 919 (4.6)	15 716 (8.3)	1 794 005 (4.7)	943 (9.8)
Cancer	1 009 589 (2.6)	13 511 (7.2)	1 022 504 (2.7)	903 (9.4)
Cardiovascular disease	10 185 676 (26.6)	90 337 (48.0)	10 271 908 (26.7)	6154 (63.8)
Coronary artery disease	1 462 550 (3.8)	16 808 (8.9)	1 478 333 (3.8)	1296 (13.4)
Cerebrovascular disease	812 338 (2.1)	9453 (5.0)	821 102 (2.1)	837 (8.7)
Chronic kidney disease	1 105 202 (2.9)	13 961 (7.4)	1 118 120 (2.9)	1258 (13.0)
Chronic lung disease	2 361 349 (6.2)	22 959 (12.2)	2 383 203 (6.2)	1520 (15.8)
Congestive heart failure	722 391 (1.9)	8681 (4.6)	730 453 (1.9)	731 (7.6)
Chronic obstructive pulmonary disease	597 688 (1.6)	7870 (4.2)	605 023 (1.6)	636 (6.6)
Dementia	343 019 (0.9)	3658 (1.9)	346 372 (0.9)	345 (3.6)
Diabetes	1 713 012 (4.5)	18 230 (9.7)	1 730 006 (4.5)	1520 (15.8)
Hypertension	8 892 597 (23.2)	79 043 (42.0)	8 967 833 (23.3)	5541 (57.4)
Liver disease	780 442 (2.0)	7727 (4.1)	787 756 (2.0)	534 (5.5)
Obesity	2 707 347 (7.1)	22 153 (11.8)	2 728 538 (7.1)	1407 (14.6)
Pregnancy	690 997 (1.8)	1763 (0.9)	692 764 (1.8)	38 (0.4)
Psoriatic arthritis	62 902 (0.2)	778 (0.4)	63 654 (0.2)	44 (0.5)
Rheumatoid arthritis	219 547 (0.6)	3885 (2.1)	223 175 (0.6)	328 (3.4)
Tobacco use/smoking	3 280 304 (8.6)	27 108 (14.4)	3 306 196 (8.6)	1722 (17.8)
Autoimmune conditions	688 401 (1.8)	9545 (5.1)	697 444 (1.8)	724 (7.5)
Immunocompromised^b^				
Any	424 727 (1.1)	6282 (3.3)	430 628 (1.1)	500 (5.2)
HIV infection	168 399 (0.4)	2233 (1.2)	170 472 (0.4)	187 (1.9)
Organ transplant or immunosuppressive therapy	51 485 (0.1)	1146 (0.6)	52 588 (0.1)	87 (0.9)
Blood transplant/stem cell transplant	35 881 (0.1)	974 (0.5)	36 792 (0.1)	87 (0.9)
Primary immunodeficiency	33 166 (0.1)	471 (0.3)	33 610 (0.1)	34 (0.4)
Active malignancy	340 323 (0.9)	3525 (1.9)	343 684 (0.9)	237 (2.5)

Data are presented as No. (%) unless noted otherwise.

^a^The Quan–Charlson Comorbidity Index score includes the following clinical conditions: myocardial infarction, congestive heart failure, peripheral vascular disease, cerebrovascular disease, dementia, chronic pulmonary disease, rheumatologic disease, peptic ulcer disease, mild liver disease, diabetes, diabetes with chronic complications, hemiplegia or paraplegia, renal disease, any malignancy (including leukemia or lymphoma), moderate or severe liver disease, metastatic solid tumor, and AIDS. Comorbidity weights were taken from the original Charlson Comorbidity Index by Charlson et al [8].

^b^Immunocompromised status defined according to Polinski et al [9].

**Figure 1.**
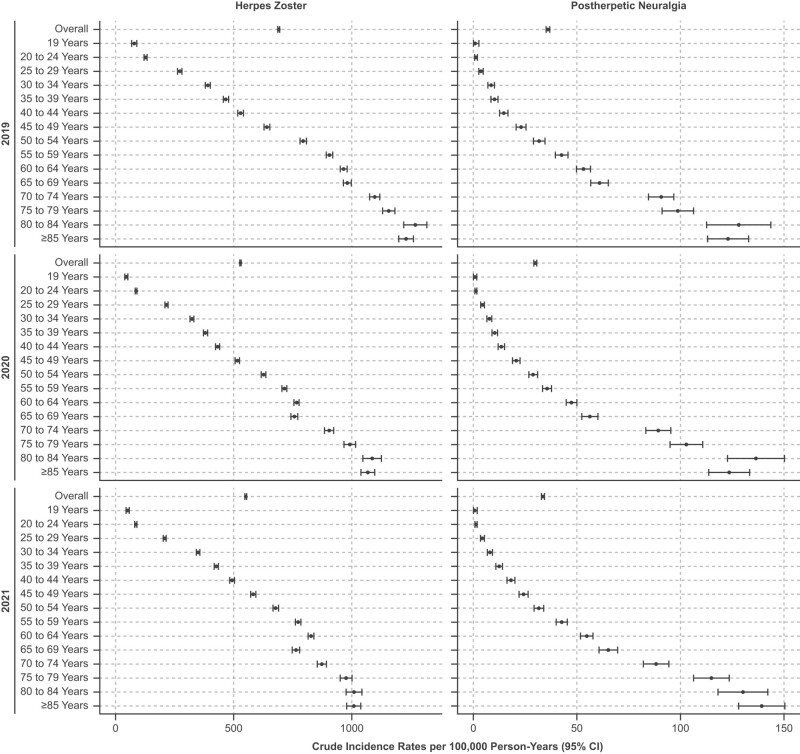
Crude incidence rates of herpes zoster and postherpetic neuralgia overall and by age (2019–2021).

A total of 188 244 HZ cases were observed in 2019, 155 668 in 2020, and 141 490 in 2021 (Supplementary Table 4). The crude and standardized incidence rates of HZ per 100 000 person-years, respectively, were 674.58 (95% CI, 671.53–677.63) and 685.48 (95% CI, 682.25–688.73) in 2019, 519.09 (95% CI, 516.51–521.67) and 542.11 (95% CI, 539.28–544.96) in 2020, and 542.68 (95% CI, 539.86–545.51) and 561.48 (95% CI, 558.47–564.50) in 2021 (Figure 1). Incidence rates of HZ increased with age. The crude HZ incidence rate per 100 000 person-years for males was 529.02 (95% CI, 525.07–532.97) in 2019, 402.74 (95% CI, 399.40–406.08) in 2020, and 424.65 (95% CI, 420.93–428.37) in 2021 (Supplementary Figure 3, Supplementary Table 5). The crude HZ incidence rate per 100 000 person-years for females was 802.16 (95% CI, 797.60–806.71) in 2019, 619.18 (95% CI, 615.34–623.03) in 2020, and 640.08 (95% CI, 635.93–644.23) in 2021. The crude incidence rate of HZ per 100 000 person-years for those who were immunocompromised was 1930.43 (95% CI, 1882.69–1978.16) in 2019, 1373.11 (95% CI, 1338.70–1407.52) in 2020, and 1355.61 (95% CI, 1322.58–1388.65) in 2021 (Supplementary Figure 4, Supplementary Table 6). Among the immunocompromised cohort, the highest incidence rates were observed in those with a history of blood/stem cell transplantation.

A total of 9649 PHN cases were observed in 2019, 8904 in 2020, and 8778 in 2021 (Supplementary Table 4). Crude and standardized incidence rates of PHN per 100 000 person-years, respectively, were 34.37 (95% CI, 33.78–35.16) and 37.53 (95% CI, 36.74–38.34) in 2019, 29.60 (95% CI, 28.99–30.22) and 34.63 (95% CI, 33.86–35.41) in 2020, and 33.56 (95% CI, 32.86–34.26) and 38.13 (95% CI, 37.31–38.97) in 2021 (Figure 1). Incidence rates of PHN increased with age. The crude PHN incidence rate per 100 000 person-years for males was 26.24 (95% CI, 25.37–27.12) in 2019, 22.51 (95% CI, 21.72–23.30) in 2020, and 25.74 (95% CI, 24.83–26.66) in 2021 (Supplementary Figure 3, Supplementary Table 5). The crude PHN incidence rate per 100 000 person-years for females was 41.67 (95% CI, 40.64–42.71) in 2019, 35.70 (95% CI, 34.78–36.62) in 2020, and 40.00 (95% CI, 38.97–41.04) in 2021. The crude incidence rate of PHN per 100 000 person-years for the immunocompromised cohort was 152.25 (95% CI, 138.90–165.59) in 2019, 116.51 (95% CI, 106.53–126.50) in 2020, and 124.74 (95% CI, 114.76–134.72) in 2021 (Supplementary Figure 4, Supplementary Table 6). Among those who were immunocompromised, the highest incidence rates were observed in those with a history of blood/stem cell transplantation.

### HZ Vaccination Uptake

**Figure 2.**
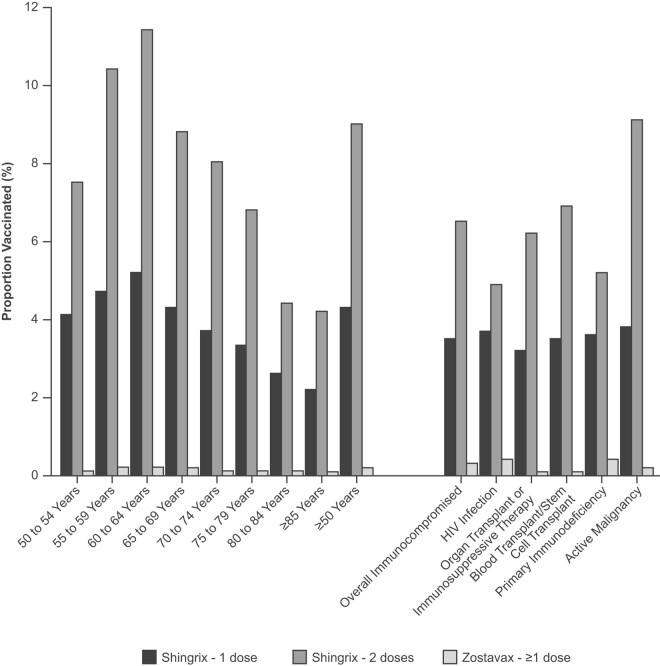
Prevalence of herpes zoster vaccination (1 May 2019–31 May 2022): 1 or 2 doses of recombinant zoster vaccine (Shingrix), as well as 1 dose of zoster vaccine live (Zostavax), by age and immunocompromised status.

Among those aged ≥50 years in the analytic cohort meeting inclusion/exclusion criteria (n = 25 286 865), 4.3% (n = 1 087 066) received 1 dose of Shingrix, 9.0% (n = 2 273 827) 2 doses of Shingrix, and 0.2% (n = 39 535) 1 dose of Zostavax from 1 January 2019 to 31 May 2022 (Figure 2).

## DISCUSSION

In this observational cohort study from 2019 to 2021, the standardized annual incidence rates of HZ ranged from 542 to 685 per 100 000 person-years, and the standardized annual incidence rates of PHN ranged from 35 to 38 per 100 000 person-years. For HZ and PHN, a gradual increase in the incidence rate was observed with increasing age, and incidence rates were highest among females, older adults, and individuals who were immunocompromised. In addition, HZ vaccine uptake from 2019 to 2022 was low overall. These results provide contemporary claims-based evidence on the disease burden of HZ and PHN and HZ vaccine uptake in the United States.

Our results should be interpreted in the context of published literature. Using the Optum database from 1994 to 2018, Thompson et al reported a crude incidence rate of HZ of 503 per 100 000 person-years overall, 413 per 100 000 person-years for males, and 589 per 100 000 person-years for females [5]. In addition, a population-based study of adults from 1 January 1996 to 15 October 2005 based on medical record review to define HZ reported a crude incidence rate of 340 per 100 000 person-years overall, 280 per 100 000 person-years for males, and 390 per 100 000 person-years in females [3]. In our claims-based study from 2019 to 2021, we found a slightly higher crude incidence rate of HZ. Moreover, Thompson et al estimated the crude incidence rate of PHN to be 65 per 100 000 person-years overall, 49 per 100 000 person-years for males, and 81 per 100 000 person-years for females. In comparison, we found a lower incidence rate of PHN likely because we defined PHN using 2 *ICD-10* codes (B02.22, postherpetic trigeminal neuralgia; B02.23, postherpetic polyneuropathy), whereas Thompson et al used a broader measurement approach, including additional *ICD-10* codes (B02.21, postherpetic geniculate ganglionitis; B02.24, postherpetic myelitis; B02.29, other postherpetic nervous system involvement). We also observed a decrease in incidence rates of HZ and PHN from 2019 to 2020, possibly due to reduced health care utilization during the COVID-19 pandemic. The contribution of the availability and uptake of Shingrix, a newer and more effective vaccine, to the decrease in HZ incidence rates is uncertain from our data.

The strengths of this study include a large and representative sample size across the United States. Due to the large sample size, we were able to estimate incidence rates among key subgroups, such as age, sex, and immunocompromised status. Additionally, the study includes several years of recent data, which allowed us to examine the potential impact of the COVID-19 pandemic on HZ and PHN incidence. The use of claims data represents 1 key limitation of this study, as only diagnosed cases of HZ and PHN were evaluated, as well as HZ vaccinations for which a claim was filed; thus, these outcomes may be underestimated. In this study, we did not estimate the proportion of patients with HZ and PHN. Patients who are diagnosed with PHN may not have sought care for HZ or may have had an HZ diagnosis before the start of the study period; therefore, to fully capture patients with PHN, we did not require a prior HZ diagnosis in our analysis. The vaccination analyses assessed prevalence from 1 January 2019 to 31 May 2022. Without vaccination data prior to this period, we could not draw conclusions regarding the potential impact of the COVID-19 pandemic on HZ vaccination rates. Additionally, some individuals may have received an HZ vaccination prior to or following the study period, resulting in underascertainment of complete vaccination. Finally, the data set used for this study does not include race or ethnicity; as such, we were not able to assess outcomes stratified by these variables.

In conclusion, HZ and PHN remain a substantial burden on US adults and were more common among females in late to older adulthood and in those who were immunocompromised. There is a need for enhanced efforts to increase vaccination against HZ.

## Supplementary Material

ofae211_Supplementary_Data

## Supplementary Data

Supplementary materials are available at *Open Forum Infectious Diseases* online. Consisting of data provided by the authors to benefit the reader, the posted materials are not copyedited and are the sole responsibility of the authors, so questions or comments should be addressed to the corresponding author.

## Notes

***Author contributions.*** Z. A. M., P. J., I. E., and E. V. contributed to the study concept and design. Z. A. M., P. J., and I. E. contributed to the acquisition of subjects and/or data. All authors contributed to the analysis and interpretation of data and the preparation of manuscript.

***Data-sharing statement.*** Individual-level data reported in this study are not publicly shared. Upon request and subject to review, Aetion, Inc, may provide the deidentified aggregate-level data that support the findings of this study. Deidentified data (including participant data as applicable) may be shared upon approval of an analysis proposal and a signed data access agreement.

***Patient consent statement.*** This noninterventional retrospective study based on an anonymized claims database certified compliant with the Health Insurance Portability and Accountability Act was deemed exempt from full review by the WIRB-Copernicus Group institutional review board. This study did not include factors necessitating patient consent.

***Presentation of work.*** An abstract based on this work was presented at IDWeek, 11–15 October 2023.

***Financial support*.** This work was supported by Moderna, Inc.
