# Supplementary material for: Incidence of Herpes Zoster and Postherpetic Neuralgia and Herpes Zoster Vaccination Uptake in a US Administrative Claims Database
Source: Open Forum Infect Dis. 2024 Apr 16;11(5):ofae211. doi: 10.1093/ofid/ofae211 (PMC11083623; doi:10.1093/ofid/ofae211)
Supplement: ofae211_Supplementary_Data [file ofae211_supplementary_data.docx]

**SUPPLEMENTAL MATERIAL**

**Table of Contents**

**Supplementary Table S1.** Codes for herpes zoster, postherpetic neuralgia, and herpes zoster vaccinations.

**Supplementary Table S2.** Baseline characteristics of patients with incident herpes zoster and postherpetic neuralgia (2020).

**Supplementary Table S3.** Baseline characteristics of patients with incident herpes zoster and postherpetic neuralgia (2021).

**Supplementary Table S4.** Crude incidence rates of herpes zoster and postherpetic neuralgia, overall and by age (2019, 2020, 2021).

**Supplementary Table S5.** Crude incidence rates of herpes zoster and postherpetic neuralgia, by sex (2019, 2020, 2021).

**Supplementary Table S6.** Crude incidence rates of herpes zoster and postherpetic neuralgia, by immunocompromised status (2019, 2020, 2021).

**Supplementary Figure S1.** Study design diagram: Incident herpes zoster and postherpetic neuralgia.

**Supplementary Figure S2.** Herpes zoster vaccination study definitions.

**Supplementary Figure S3.** Crude incidence rates of herpes zoster and postherpetic neuralgia overall and by sex (2019, 2020, 2021).

**Supplementary Figure S4.** Crude incidence rates of herpes zoster and postherpetic neuralgia overall and by immunocompromised status (2019, 2020, 2021).

**Supplementary Table S1.** Codes for herpes zoster, postherpetic neuralgia, and herpes zoster vaccinations.

| **Variable** | **Coding system** | **Codes/algorithm** |
| --- | --- | --- |
| **Outcomes** | | |
| Herpes zoster | ICD-10 | Evidence of the following diagnosis codes (one or more) in any position, in any clinical setting:  B02.9 - Zoster without complications  B02.8 - Zoster with other complications  B02.39 - Other herpes zoster eye disease  B02.30 - Zoster ocular disease, unspecified  B02.33 - Zoster keratitis  B02.7 - Disseminated zoster  B02.32 - Zoster iridocyclitis  B02.31 - Zoster conjunctivitis  B02.1 - Zoster meningitis  B02.0 - Zoster encephalitis  B02.34 - Zoster scleritis  B02.21 - Postherpetic geniculate ganglionitis  B02.24 - Postherpetic myelitis  B02.29 - Other postherpetic nervous system involvement  B02.2 - Zoster with other nervous system involvement  B02.3 - Zoster ocular disease  B02 - Zoster [herpes zoster] |
| Postherpetic neuralgia | ICD-10 | Evidence of the following diagnosis codes (one or more) in any position, in any clinical setting:  B02.22 - Postherpetic trigeminal neuralgia  B02.23 - Postherpetic polyneuropathy |
| **Vaccinations** | | |
| Shingrix vaccination  (1 dose) | CPT, brand name | ≥1 CPT codes in any position:  90750  OR  ≥1 pharmacy claims for the following:  Shingrix |
| Full Shingrix vaccination regimen  (2 doses) | CPT, brand name | Evidence of 2 doses of Shingrix vaccination, where the second occurs 30 to 210 days after the first dose within the same year |
| Zostavax vaccination | CPT, brand name | ≥1 CPT codes in any position:  90736  OR  ≥1 pharmacy claims for the following:  Zostavax |

Abbreviations: CT, Current Procedural Terminology; ICD, International Classification of Diseases.

**Supplementary Table S2.** Baseline characteristics of patients with incident herpes zoster or postherpetic neuralgia (2020).

|  | ***N* = 35,783,632** | | ***N* = 35,799,297** | |
| --- | --- | --- | --- | --- |
|  | **No herpes zoster *n* = 35,627,964** | **Herpes zoster *n* = 155,668** | **No postherpetic neuralgia *n* = 35,790,393** | **Postherpetic neuralgia *n* = 8,904** |
| **Characteristic** | - | - | - | - |
| **Age, mean (SD), years** | 45.92 (17.09) | 55.46 (15.57) | 45.96 (17.09) | 62.84 (15.03) |
| **Age categories, *n* (%), years** | | | | |
| 19 | 1,238,963 (3.5) | 377 (0.2) | 1,239,388 (3.5) | 6 (0.1) |
| 20–24 | 3,120,471 (8.8) | 2,254 (1.4) | 3,122,926 (8.7) | 33 (0.4) |
| 25–29 | 3,236,102 (9.1) | 5,442 (3.5) | 3,241,878 (9.1) | 105 (1.2) |
| 30–34 | 3,243,514 (9.1) | 8,459 (5.4) | 3,252,535 (9.1) | 204 (2.3) |
| 35–39 | 3,230,482 (9.1) | 10,191 (6.5) | 3,241,440 (9.1) | 286 (3.2) |
| 40–44 | 3,038,625 (8.5) | 11,056 (7.1) | 3,050,460 (8.5) | 341 (3.8) |
| 45–49 | 3,186,671 (8.9) | 14,054 (9.0) | 3,201,658 (8.9) | 560 (6.3) |
| 50–54 | 3,342,760 (9.4) | 18,196 (11.7) | 3,362,009 (9.4) | 855 (9.6) |
| 55–59 | 3,669,630 (10.3) | 23,047 (14.8) | 3,694,021 (10.3) | 1,181 (13.3) |
| 60–64 | 3,486,480 (9.8) | 23,481 (15.1) | 3,510,997 (9.8) | 1,475 (16.6) |
| 65–69 | 1,928,071 (5.4) | 12,766 (8.2) | 1,941,153 (5.4) | 998 (11.2) |
| 70–74 | 1,170,093 (3.3) | 9,646 (6.2) | 1,179,603 (3.3) | 963 (10.8) |
| 75–79 | 782,157 (2.2) | 7,180 (4.6) | 789,215 (2.2) | 750 (8.4) |
| 80–84 | 345,225 (1.0) | 3,480 (2.2) | 348,513 (1.0) | 431 (4.8) |
| ≥85 | 608,720 (1.7) | 6,039 (3.9) | 614,597 (1.7) | 716 (8.0) |
| **Sex, *n* (%)** | | | | |
| Male | 16,494,239 (46.3) | 55,854 (35.9) | 16,553,215 (46.3) | 3,129 (35.1) |
| Female | 19,133,725 (53.7) | 99,814 (64.1) | 19,237,178 (53.7) | 5,775 (64.9) |
| **Payer type, *n* (%)** | | | | |
| Commercial | 22,728,810 (63.8) | 93,935 (60.3) | 22,831,346 (63.8) | 4,002 (44.9) |
| Medicaid | 3,126,604 (8.8) | 27,222 (17.5) | 3,153,347 (8.8) | 2,833 (31.8) |
| Medicare | 10,758,651 (30.2) | 39,942 (25.7) | 10,796,943 (30.2) | 2,547 (28.6) |
| Other/Unknown | 312,326 (0.9) | 1,222 (0.8) | 313,639 (0.9) | 51 (0.6) |
| **Quan-Charlson Comorbidity Index score, *n* (%)^a^** | | | | |
| 0 | 28,755,333 (80.7) | 97,105 (62.4) | 28,859,369 (80.6) | 4,162 (46.7) |
| 1 | 3,297,563 (9.3) | 22,325 (14.3) | 3,320,472 (9.3) | 1,485 (16.7) |
| ≥2 | 3,575,068 (10.0) | 36,238 (23.3) | 3,610,552 (10.1) | 3,257 (36.6) |
| **Comorbidities, *n* (%)** | | | | |
| Alcohol use | 732,877 (2.1) | 3,845 (2.5) | 736,660 (2.1) | 275 (3.1) |
| Arrhythmia | 2,664,435 (7.5) | 24,244 (15.6) | 2,688,468 (7.5) | 1,976 (22.2) |
| Asthma | 2,163,587 (6.1) | 16,711 (10.7) | 2,180,328 (6.1) | 1,177 (13.2) |
| Cancer | 1,082,630 (3.0) | 11,919 (7.7) | 1,094,639 (3.1) | 967 (10.9) |
| Cardiovascular disease | 10,700,976 (30.0) | 82,676 (53.1) | 10,784,592 (30.1) | 6,305 (70.8) |
| Coronary artery disease | 1,577,328 (4.4) | 16,284 (10.5) | 1,593,175 (4.5) | 1,554 (17.5) |
| Cerebrovascular disease | 962,529 (2.7) | 10,274 (6.6) | 972,386 (2.7) | 1,021 (11.5) |
| Chronic kidney disease | 1,207,256 (3.4) | 13,335 (8.6) | 1,220,089 (3.4) | 1,359 (15.3) |
| Chronic lung disease | 2,838,238 (8.0) | 24,116 (15.5) | 2,862,294 (8.0) | 1,780 (20.0) |
| Congestive heart failure | 814,021 (2.3) | 8,773 (5.6) | 822,423 (2.3) | 919 (10.3) |
| COPD | 731,757 (2.1) | 8,719 (5.6) | 740,225 (2.1) | 781 (8.8) |
| Dementia | 359,030 (1.0) | 3,674 (2.4) | 362,556 (1.0) | 337 (3.8) |
| Diabetes | 1,825,002 (5.1) | 17,431 (11.2) | 1,841,931 (5.1) | 1,634 (18.4) |
| Hypertension | 9,225,837 (25.9) | 71,677 (46.0) | 9,297,991 (26.0) | 5,644 (63.4) |
| Liver disease | 1,011,385 (2.8) | 8,918 (5.7) | 1,020,169 (2.9) | 672 (7.5) |
| Obesity | 3,553,660 (10.0) | 26,480 (17.0) | 3,580,094 (10.0) | 1,903 (21.4) |
| Pregnancy | 1,043,615 (2.9) | 2,608 (1.7) | 1,046,341 (2.9) | 59 (0.7) |
| Psoriatic arthritis | 72,953 (0.2) | 804 (0.5) | 73,783 (0.2) | 51 (0.6) |
| Rheumatoid arthritis | 254,168 (0.7) | 4,029 (2.6) | 258,090 (0.7) | 363 (4.1) |
| Tobacco use/smoking | 3,975,233 (11.2) | 29,259 (18.8) | 4,004,361 (11.2) | 2,101 (23.6) |
| Autoimmune conditions | 809,304 (2.3) | 10,155 (6.5) | 819,395 (2.3) | 814 (9.1) |
| **Immunocompromised^b^** | | | | |
| Any | 508,729 (1.4) | 6,117 (3.9) | 514,761 (1.4) | 523 (5.9) |
| HIV infection | 181,621 (0.5) | 1,976 (1.3) | 183,501 (0.5) | 172 (1.9) |
| Organ transplant or  immunosuppressive therapy | 56,672 (0.2) | 1,144 (0.7) | 57,821 (0.2) | 91 (1.0) |
| Blood transplant/stem cell  transplant | 48,806 (0.1) | 1,080 (0.7) | 49,888 (0.1) | 85 (1.0) |
| Primary immunodeficiency | 43,227 (0.1) | 512 (0.3) | 43,734 (0.1) | 47 (0.5) |
| Active malignancy | 371,107 (1.0) | 3,105 (2.0) | 374,249 (1.0) | 258 (2.9) |

Abbreviations: COPD, chronic obstructive pulmonary disease; HIV, human immunodeficiency virus; SD, standard deviation.

^a^The Quan-Charlson Comorbidity Index score includes the following clinical conditions: myocardial infarction, congestive heart failure, peripheral vascular disease, cerebrovascular disease, dementia, chronic pulmonary disease, rheumatologic disease, peptic ulcer disease, mild liver disease, diabetes, diabetes with chronic complications, hemiplegia or paraplegia, renal disease, any malignancy (including leukemia or lymphoma), moderate or severe liver disease, metastatic solid tumor, and AIDS. Comorbidity weights were taken from the original Charlson Comorbidity Index by Charlson et al.[1]

^b^Immunocompromised status defined according to Polinski et al.[2]

**Supplementary Table S3.** Baseline characteristics of patients with incident herpes zoster or postherpetic neuralgia (2021).

|  | ***N* = 29,733,319** | | ***N* = 29,752,654** | |
| --- | --- | --- | --- | --- |
|  | **No herpes zoster *n* = 29,591,829** | **Herpes zoster *n* = 141,490** | **No postherpetic neuralgia *n* = 29,743,876** | **Postherpetic neuralgia *n* = 8778** |
| **Characteristic** | - | - | - | - |
| **Age, mean (SD), years** | 46.08 (17.68) | 55.31 (15.75) | 46.13 (17.68) | 63.39 (15.36) |
| **Age, *n* (%), years** | | | | |
| 19 | 1,199,069 (4.1) | 411 (0.3) | 1,199,517 (4.0) | 6 (0.1) |
| 20–24 | 2,641,985 (8.9) | 2,030 (1.4) | 2,644,205 (8.9) | 30 (0.3) |
| 25–29 | 2,722,705 (9.2) | 4,790 (3.4) | 2,727,916 (9.2) | 103 (1.2) |
| 30–34 | 2,707,855 (9.2) | 8,147 (5.8) | 2,716,739 (9.1) | 185 (2.1) |
| 35–39 | 2,612,957 (8.8) | 9,877 (7.0) | 2,623,740 (8.8) | 287 (3.3) |
| 40–44 | 2,464,107 (8.3) | 10,758 (7.6) | 2,475,802 (8.3) | 387 (4.4) |
| 45–49 | 2,424,815 (8.2) | 12,699 (9.0) | 2,438,589 (8.2) | 531 (6.0) |
| 50–54 | 2,627,733 (8.9) | 16,005 (11.3) | 2,645,192 (8.9) | 754 (8.6) |
| 55–59 | 2,868,607 (9.7) | 19,947 (14.1) | 2,890,397 (9.7) | 1,134 (12.9) |
| 60–64 | 2,908,411 (9.8) | 21,477 (15.2) | 2,931,640 (9.9) | 1,426 (16.2) |
| 65–69 | 1,609,148 (5.4) | 11,028 (7.8) | 1,621,061 (5.5) | 978 (11.1) |
| 70–74 | 1,115,977 (3.8) | 8,856 (6.3) | 1,125,146 (3.8) | 920 (10.5) |
| 75–79 | 741,292 (2.5) | 6,577 (4.6) | 747,972 (2.5) | 797 (9.1) |
| 80–84 | 418,599 (1.4) | 3,912 (2.8) | 422,440 (1.4) | 527 (6.0) |
| ≥85 | 528,569 (1.8) | 4,976 (3.5) | 533,520 (1.8) | 713 (8.1) |
| **Sex, *n* (%)** | | | | |
| Male | 13,367,716 (45.2) | 50,055 (35.4) | 13,422,285 (45.1) | 3,042 (34.7) |
| Female | 16,224,113 (54.8) | 91,435 (64.6) | 16,321,591 (54.9) | 5,736 (65.3) |
| **Payer type, *n* (%)** | | | | |
| Commercial | 16,653,919 (56.3) | 79,424 (56.1) | 16,744,832 (56.3) | 3,639 (41.5) |
| Medicaid | 2,898,829 (9.8) | 24,417 (17.3) | 2,923,679 (9.8) | 2,865 (32.6) |
| Medicare | 10,601,425 (35.8) | 41,427 (29.3) | 10,641,162 (35.8) | 2,656 (30.3) |
| Other/Unknown | 295,159 (1.0) | 1,440 (1.0) | 296,778 (1.0) | 69 (0.8) |
| **Quan-Charlson Comorbidity Index score, *n* (%)^a^** | | | | |
| 0 | 23,756,287 (80.3) | 88,474 (62.5) | 23,854,361 (80.2) | 4,039 (46.0) |
| 1 | 2,728,015 (9.2) | 20,136 (14.2) | 2,749,099 (9.2) | 1,496 (17.0) |
| ≥2 | 3,107,527 (10.5) | 32,880 (23.2) | 3,140,416 (10.6) | 3,243 (36.9) |
| **Comorbidities, *n* (%)** | | | | |
| Alcohol use | 828,374 (2.8) | 4,462 (3.2) | 832,896 (2.8) | 326 (3.7) |
| Arrhythmia | 2,904,145 (9.8) | 25,951 (18.3) | 2,930,869 (9.9) | 2,198 (25.0) |
| Asthma | 2,253,516 (7.6) | 17,521 (12.4) | 2,271,560 (7.6) | 1,371 (15.6) |
| Cancer | 1,043,395 (3.5) | 11,597 (8.2) | 1,055,469 (3.5) | 1,051 (12.0) |
| Cardiovascular disease | 9,992,249 (33.8) | 78,878 (55.7) | 10,074,746 (33.9) | 6,434 (73.3) |
| Coronary artery disease | 1,598,321 (5.4) | 16,047 (11.3) | 1,614,537 (5.4) | 1,604 (18.3) |
| Cerebrovascular disease | 1,036,902 (3.5) | 10,753 (7.6) | 1,047,575 (3.5) | 1,171 (13.3) |
| Chronic kidney disease | 1,224,557 (4.1) | 12,837 (9.1) | 1,237,285 (4.2) | 1,437 (16.4) |
| Chronic lung disease | 2,927,939 (9.9) | 24,453 (17.3) | 2,953,034 (9.9) | 2,033 (23.2) |
| Congestive heart failure | 874,606 (3.0) | 8,988 (6.4) | 883,480 (3.0) | 981 (11.2) |
| COPD | 769,923 (2.6) | 8,595 (6.1) | 778,528 (2.6) | 824 (9.4) |
| Dementia | 382,809 (1.3) | 3,678 (2.6) | 386,335 (1.3) | 459 (5.2) |
| Diabetes | 1,812,526 (6.1) | 17,211 (12.2) | 1,829,519 (6.2) | 1,776 (20.2) |
| Hypertension | 8,563,822 (28.9) | 68,077 (48.1) | 8,634,577 (29.0) | 5,740 (65.4) |
| Liver disease | 1,128,190 (3.8) | 10,095 (7.1) | 1,138,326 (3.8) | 844 (9.6) |
| Obesity | 3,853,029 (13.0) | 29,226 (20.7) | 3,883,270 (13.1) | 2,179 (24.8) |
| Pregnancy | 1,318,287 (4.5) | 3,770 (2.7) | 1,322,302 (4.4) | 97 (1.1) |
| Psoriatic arthritis | 71,856 (0.2) | 827 (0.6) | 72,748 (0.2) | 62 (0.7) |
| Rheumatoid arthritis | 265,614 (0.9) | 4,144 (2.9) | 269,777 (0.9) | 415 (4.7) |
| Tobacco use/smoking | 4,196,906 (14.2) | 30,744 (21.7) | 4,228,591 (14.2) | 2,311 (26.3) |
| Autoimmune conditions | 820,924 (2.8) | 10,287 (7.3) | 831,505 (2.8) | 913 (10.4) |
| **Immunocompromised^b^** | | | | |
| Any | 530,735 (1.8) | 6,469 (4.6) | 537,290 (1.8) | 600 (6.8) |
| HIV infection | 175,365 (0.6) | 1,932 (1.4) | 177,241 (0.6) | 155 (1.8) |
| Organ transplant or  immunosuppressive therapy | 62,816 (0.2) | 1,321 (0.9) | 64,155 (0.2) | 130 (1.5) |
| Blood transplant/stem cell  transplant | 70,097 (0.2) | 1,507 (1.1) | 71,616 (0.2) | 158 (1.8) |
| Primary immunodeficiency | 47,940 (0.2) | 541 (0.4) | 48,493 (0.2) | 53 (0.6) |
| Active malignancy | 357,249 (1.2) | 3,090 (2.2) | 360,494 (1.2) | 291 (3.3) |

Abbreviations: COPD, chronic obstructive pulmonary disease; HIV, human immunodeficiency virus; SD, standard deviation.

^a^The Quan-Charlson Comorbidity Index score includes the following clinical conditions: myocardial infarction, congestive heart failure, peripheral vascular disease, cerebrovascular disease, dementia, chronic pulmonary disease, rheumatologic disease, peptic ulcer disease, mild liver disease, diabetes, diabetes with chronic complications, hemiplegia or paraplegia, renal disease, any malignancy (including leukemia or lymphoma), moderate or severe liver disease, metastatic solid tumor, and AIDS. Comorbidity weights were taken from the original Charlson Comorbidity Index by Charlson et al.[1]

^b^Immunocompromised status defined according to Polinski et al.[2]

**Supplementary Table S4.** Crude incidence rates of herpes zoster and postherpetic neuralgia, overall and by age (2019, 2020, 2021).

|  | **2019** | | | **2020** | | | **2021** | | |
| --- | --- | --- | --- | --- | --- | --- | --- | --- | --- |
|  | ***N* person-years** | ***N* patients with an event** | **Crude incidence rate per 100,000 person-years (95% CI)** | ***N* person-years** | ***N* patients with an event** | **Crude incidence rate per 100,000 person-years**  **(95% CI)** | ***N* person-years** | ***N* patients with an event** | **Crude incidence rate per 100,000 person-years**  **(95% CI)** |
| **Herpes Zoster** | | | | | | | | | |
| Overall | 27,905,401.59 | 188,244 | 674.58  (671.53–677.63) | 29,988,699.24 | 155,668 | 519.09  (516.51–521.67) | 26,072,277.14 | 141,490 | 542.68  (539.86–545.51) |
| **Age, years** | | | | | | | | | |
| 19 | 723,659.75 | 522 | 72.13  (65.95–78.32) | 815,414.52 | 377 | 46.23  (41.57–50.90) | 832,814.11 | 411 | 49.35  (44.58–54.12) |
| 20–24 | 2,315,943.78 | 3007 | 129.84  (125.20–134.48) | 2,588,092.19 | 2254 | 87.09  (83.50–90.69) | 2,341,126.05 | 2030 | 86.71  (82.94–90.48) |
| 25–29 | 2,283,882.87 | 6590 | 288.54  (281.58–295.51) | 2,535,857.54 | 5442 | 214.60  (208.90–220.30) | 2,324,517.66 | 4790 | 206.06  (200.23–211.90) |
| 30–34 | 2,358,215.28 | 9483 | 402.13  (394.03–410.22) | 2,632,212.76 | 8459 | 321.36  (314.52–328.21) | 2,360,772.35 | 8147 | 345.10  (337.61–352.59) |
| 35–39 | 2,429,162.17 | 11,608 | 477.86  (469.17–486.55) | 2,696,185.12 | 10,191 | 377.98  (370.64–385.32) | 2,316,906.41 | 9877 | 426.30  (417.89–434.71) |
| 40–44 | 2,303,788.20 | 12,496 | 542.41  (532.90–551.92) | 2,575,589.76 | 11,056 | 429.26  (421.26–437.26) | 2,204,254.92 | 10,758 | 488.06  (478.83–497.28) |
| 45–49 | 2,530,659.38 | 16,560 | 654.37  (644.41–664.34) | 2,741,283.61 | 14,054 | 512.68  (504.20–521.16) | 2,185,213.70 | 12,699 | 581.13  (571.03–591.24) |
| 50–54 | 2,652,454.96 | 21,356 | 805.14  (794.34–815.94) | 2,902,088.45 | 18,196 | 627.00  (617.89–636.11) | 2,370,042.28 | 16,005 | 675.30  (664.84–685.77) |
| 55–59 | 2,911,965.65 | 26,955 | 925.66  (914.61–936.71) | 3,224,754.68 | 23,047 | 714.69  (705.46–723.92) | 2,594,270.57 | 19,947 | 768.89  (758.22–779.56) |
| 60–64 | 2,834,727.28 | 28,150 | 993.04  (981.44–1004.64) | 3,019,010.85 | 23,481 | 777.77  (767.82–787.72) | 2,585,549.65 | 21,477 | 830.66  (819.55–841.76) |
| 65–69 | 1,731,963.07 | 17,017 | 982.53  (967.76–997.29) | 1,657,022.02 | 12,766 | 770.42  (757.05–783.78) | 1,418,448.63 | 11,028 | 777.47  (762.96–791.98) |
| 70–74 | 1,155,314.55 | 13,127 | 1136.23  (1116.79–1155.66) | 1,045,822.39 | 9646 | 922.34  (903.93–940.74) | 1,006,561.58 | 8856 | 879.83  (861.50–898.15) |
| 75–79 | 793,200.16 | 9561 | 1205.37  (1181.21–1229.53) | 704,535.82 | 7180 | 1019.11  (995.54–1042.68) | 671,027.38 | 6577 | 980.14  (956.45–1003.83) |
| 80–84 | 248,985.55 | 3323 | 1334.62  (1289.24–1379.99) | 308,272.04 | 3480 | 1128.87  (1091.37–1166.38) | 380,540.54 | 3912 | 1028.01 (995.80–1060.23) |
| ≥85 | 631,478.93 | 8489 | 1344.30  (1315.71–1372.90) | 542,557.48 | 6039 | 1113.06  (1084.99–1141.13) | 480,231.31 | 4976 | 1036.17 (1007.38–1064.96) |
| **Postherpetic neuralgia** | | | | | | | | | |
| Overall | 27,991,190.81 | 9649 | 34.47  (33.78–35.16) | 30,078,047.88 | 8904 | 29.60  (28.99–30.22) | 26,157,093.06 | 8778 | 33.56  (32.86–34.26) |
| **Age–year** | | | | | | | | | |
| 19 | 723,873.54 | 6 | 0.83  (0.30–1.80) | 815,597.57 | 6 | 0.74  (0.27–1.60) | 833,019.74 | 6 | 0.72  (0.26–1.57) |
| 20–24 | 2,317,294.90 | 31 | 1.34  (0.87–1.81) | 2,589,392.45 | 33 | 1.27  (0.84–1.71) | 2,342,282.23 | 30 | 1.28  (0.82–1.74) |
| 25–29 | 2,286,679.08 | 108 | 4.72  (3.83–5.61) | 2,538,782.37 | 105 | 4.14  (3.34–4.93) | 2,327,241.95 | 103 | 4.43  (3.57–5.28) |
| 30–34 | 2,362,314.91 | 218 | 9.23  (8.00–10.45) | 2,636,948.20 | 204 | 7.74  (6.67–8.80) | 2,365,522.65 | 185 | 7.82  (6.69–8.95) |
| 35–39 | 2,434,340.55 | 283 | 11.63  (10.27–12.98) | 2,702,167.93 | 286 | 10.58  (9.36–11.81) | 2,322,651.90 | 287 | 12.36  (10.93–13.79) |
| 40–44 | 2,309,298.19 | 374 | 16.20  (14.55–17.84) | 2,581,997.54 | 341 | 13.21  (11.81–14.61) | 2,210,591.67 | 387 | 17.51  (15.76–19.25) |
| 45–49 | 2,538,061.68 | 594 | 23.40  (21.52–25.29) | 2,749,432.14 | 560 | 20.37  (18.68–22.05) | 2,192,747.34 | 531 | 24.22  (22.16–26.28) |
| 50–54 | 2,662,058.29 | 875 | 32.87  (30.69–35.05) | 2,912,743.27 | 855 | 29.35  (27.39–31.32) | 2,379,768.94 | 754 | 31.68  (29.42–33.95) |
| 55–59 | 2,924,061.88 | 1311 | 44.83  (42.41–47.26) | 3,238,388.78 | 1181 | 36.47  (34.39–38.55) | 2,606,605.24 | 1134 | 43.50  (40.97–46.04) |
| 60–64 | 2,847,374.98 | 1555 | 54.61  (51.90–57.33) | 3,032,540.51 | 1475 | 48.64  (46.16–51.12) | 2,598,641.72 | 1426 | 54.87  (52.03–57.72) |
| 65–69 | 1,740,171.17 | 1138 | 65.40  (61.60–69.20) | 1,664,374.15 | 998 | 59.96  (56.24–63.68) | 1,425,223.62 | 978 | 68.62  (64.32–72.92) |
| 70–74 | 1,161,701.70 | 1083 | 93.23  (87.67–98.78) | 1,051,145.29 | 963 | 91.61  (85.83–97.40) | 1,011,871.44 | 920 | 90.92  (85.05–96.80) |
| 75–79 | 797,920.22 | 824 | 103.27  (96.22–110.32) | 708,509.48 | 750 | 105.86  (98.28–113.43) | 674,960.18 | 797 | 118.08  (109.88–126.28) |
| 80–84 | 250,553.63 | 347 | 138.49  (123.92–153.07) | 310,126.08 | 431 | 138.98  (125.86–152.10) | 382,782.05 | 527 | 137.68  (125.92–149.43) |
| ≥85 | 635,486.07 | 902 | 141.94  (132.68–151.20) | 545,902.13 | 716 | 131.16  (121.55–140.77) | 483,182.38 | 713 | 147.56  (136.73–158.39) |

**Supplementary Table S5.** Crude incidence rates of herpes zoster and postherpetic neuralgia, by sex (2019, 2020, 2021).

|  | **2019** | | | **2020** | | | **2021** | | |
| --- | --- | --- | --- | --- | --- | --- | --- | --- | --- |
|  | ***N* person-years** | ***N* patients with an event** | **Crude incidence rate per 100,000 person-years (95% CI)** | ***N* person-years** | ***N* patients with an event** | **Crude incidence rate per 100,000 person-years**  **(95% CI)** | ***N* person-years** | ***N* patients with an event** | **Crude incidence rate per 100,000 person-years**  **(95% CI)** |
| **Herpes zoster** | | | | | | | | | |
| **Sex** | | | | | | | | | |
| Male | 13,034,099.45 | 68,953 | 529.02  (525.07–532.97) | 13,868,459.33 | 55,854 | 402.74  (399.40–406.08) | 11,787,374.29 | 50,055 | 424.65  (420.93–428.37) |
| Female | 14,871,302.15 | 119,291 | 802.16 (797.60–806.71) | 16,120,239.91 | 99,814 | 619.18  (615.34–623.03) | 14,284,902.85 | 91,435 | 640.08  (635.93–644.23) |
| **Postherpetic neuralgia** | | | | | | | | | |
| **Sex** | | | | | | | | | |
| Male | 13,065,752.00 | 3429 | 26.24  (25.37–27.12) | 13,901,078.26 | 3129 | 22.51  (21.72–23.30) | 11,817,895.03 | 3042 | 25.74  (24.83–26.66) |
| Female | 14,925,438.81 | 6220 | 41.67  (40.64–42.71) | 16,176,969.62 | 5775 | 35.70  (34.78–36.62) | 14,339,198.02 | 5736 | 40.00  (38.97–41.04) |

**Supplementary Table S6.** Crude incidence rates of herpes zoster and postherpetic neuralgia, by immunocompromised status (2019, 2020, 2021).

|  | **2019** | | | **2020** | | | **2021** | | |
| --- | --- | --- | --- | --- | --- | --- | --- | --- | --- |
|  | ***N* person-years** | ***N* patients with an event** | **Crude incidence rate per 100,000 person-years (95% CI)** | ***N* person-years** | ***N* patients with an event** | **Crude incidence rate per 100,000 person-years**  **(95% CI)** | ***N* person-years** | ***N* patients with an event** | **Crude incidence rate per 100,000 person-years**  **(95% CI)** |
| **Herpes zoster** | | | | | | | | | |
| **Immunocompromised status^a^** | | | | | | | | | |
| Any | 325,420.56 | 6282 | 1930.43 (1882.69–1978.16) | 445,486.34 | 6117 | 1373.11 (1338.70–1407.52) | 477,201.78 | 6469 | 1355.61  (1322.58–1388.65) |
| HIV infection | 125,094.80 | 2233 | 1785.05 (1711.01–1859.08) | 154,334.37 | 1976 | 1280.34 (1223.89–1336.79) | 156,559.61 | 1932 | 1234.03  (1179.01–1289.06) |
| Organ transplant or  immunosuppressive therapy | 38,815.35 | 1146 | 2952.44 (2781.50–3123.38) | 50,033.87 | 1144 | 2286.45 (2153.96–2418.95) | 56,025.52 | 1321 | 2357.85  (2230.70–2485.00) |
| Blood transplant/stem  cell transplant | 27,178.92 | 974 | 3583.66 (3358.60–3808.72) | 42,897.37 | 1080 | 2517.64 (2367.49–2667.79) | 61,769.07 | 1507 | 2439.73  (2316.55–2562.91) |
| Primary  immunodeficiency | 25,758.00 | 471 | 1828.56 (1663.42–1993.70) | 38,084.64 | 512 | 1344.37 (1227.93–1460.82) | 43,443.95 | 541 | 1245.28  (1140.35–1350.22) |
| Active malignancy | 267,767.98 | 3525 | 1316.44 (1272.98–1359.90) | 331,205.84 | 3105 | 937.48 (904.51–970.46) | 322,433.27 | 3090 | 958.34  (924.55–992.13) |
| **Postherpetic neuralgia** | | | | | | | | | |
| **Immunocompromised status^a^** | | | | | | | | | |
| Any | 328,413.83 | 500 | 152.25 (138.90–165.59) | 448,883.01 | 523 | 116.51  (106.53–126.50) | 480,992.67 | 600 | 124.74  (114.76–134.72) |
| HIV infection | 126,149.31 | 187 | 148.24 (126.99–169.48) | 155,367.99 | 172 | 110.70  (94.16–127.25) | 157,610.77 | 155 | 98.34  (82.86–113.83) |
| Organ transplant or  immunosuppressive therapy | 39,363.48 | 87 | 221.02 (174.57–267.46) | 50,685.11 | 91 | 179.54  (142.65–216.43) | 56,814.81 | 130 | 228.81  (189.48–268.15) |
| Blood transplant/stem  cell transplant | 27,642.83 | 87 | 314.73 (248.59–380.86) | 43,543.29 | 85 | 195.21  (153.71–236.71) | 62,673.39 | 158 | 252.10  (212.79–291.41) |
| Primary immunodeficiency | 25,980.53 | 34 | 130.87  (86.88–174.86) | 38,362.31 | 47 | 122.52  (87.49–157.54) | 43,769.05 | 53 | 121.09  (88.49–153.69) |
| Active malignancy | 269,484.30 | 237 | 87.95  (76.75–99.14) | 332,975.02 | 258 | 77.48  (68.03–86.94) | 324,303.35 | 291 | 89.73  (79.42–100.04) |

Abbreviations: HIV, human immunodeficiency virus.
^a^Immunocompromised status defined according to Polinski et al.[2]

**Supplementary Figure S1.** Study design diagram: Incident herpes zoster and postherpetic neuralgia.

**
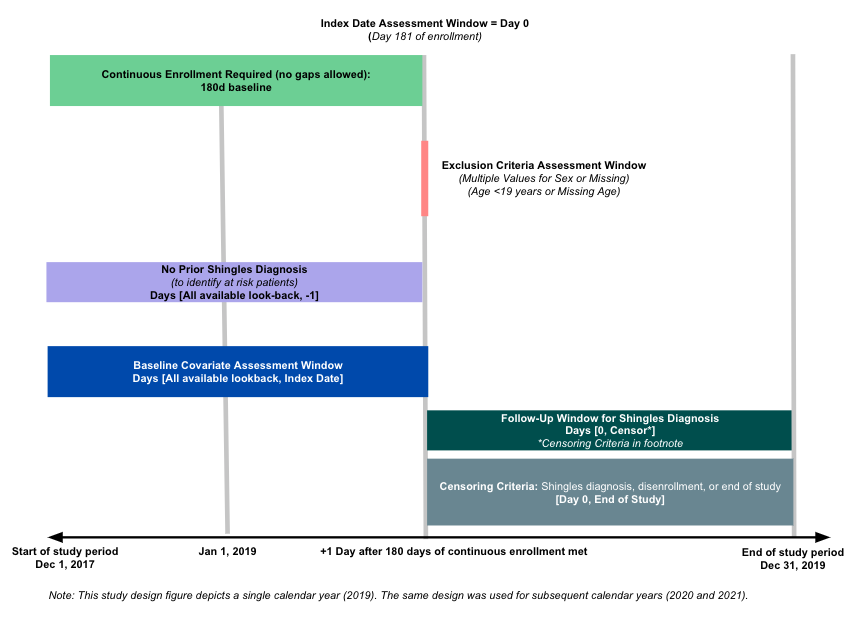
**
*Note*: The study design to examine the incidence of postherpetic neuralgia (PHN) was identical. Day 0 is defined as 1 day following the fulfillment of the 180-day baseline continuous enrollment requirement (no gaps allowed). Day 0 is the index date. The incidence analyses include a washout period to ensure no prior history of herpes zoster (shingles) (or PHN when examining the incidence of PHN).

**Supplementary Figure S2.** Herpes zoster vaccination study definitions.

**
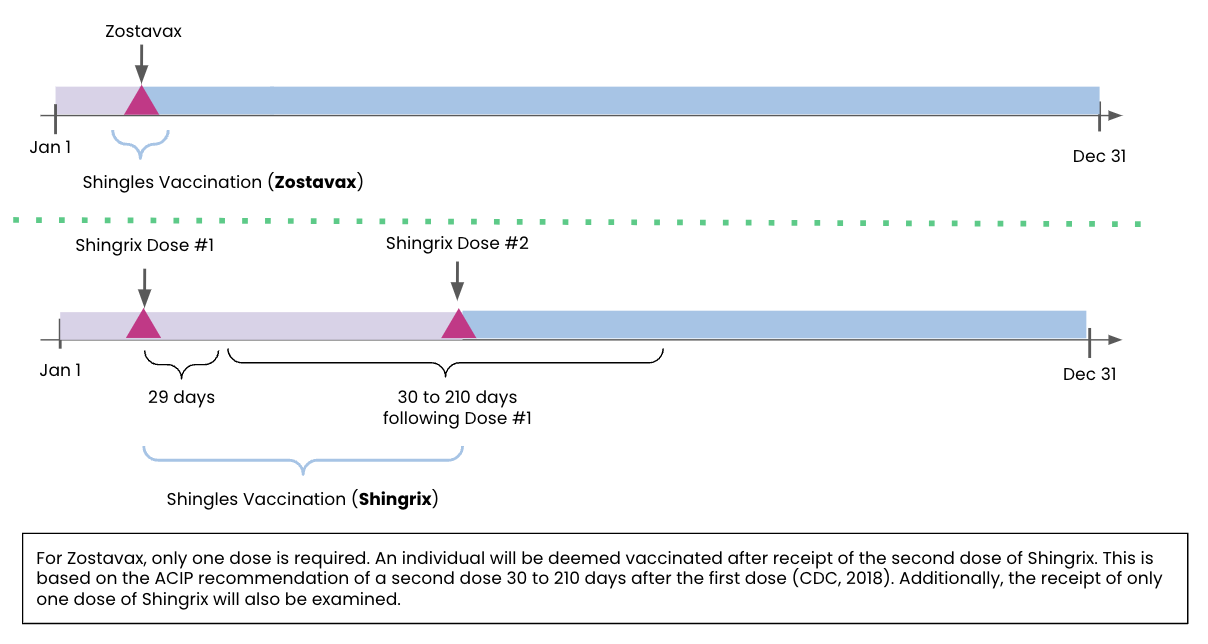
**

Abbreviations: ACIP, CDC Advisory Committee on Immunization Practices; CDC, Centers for Disease Control and Prevention.

*Note*: Zostavax requires only one dose, and Shingrix requires two doses. Individuals meeting the definition for receipt of either shingles vaccine were included in the outcome, as depicted in figure above.

**Supplementary Figure S3**. Crude incidence rates of herpes zoster and postherpetic neuralgia, overall and by sex (2019, 2020, 2021).


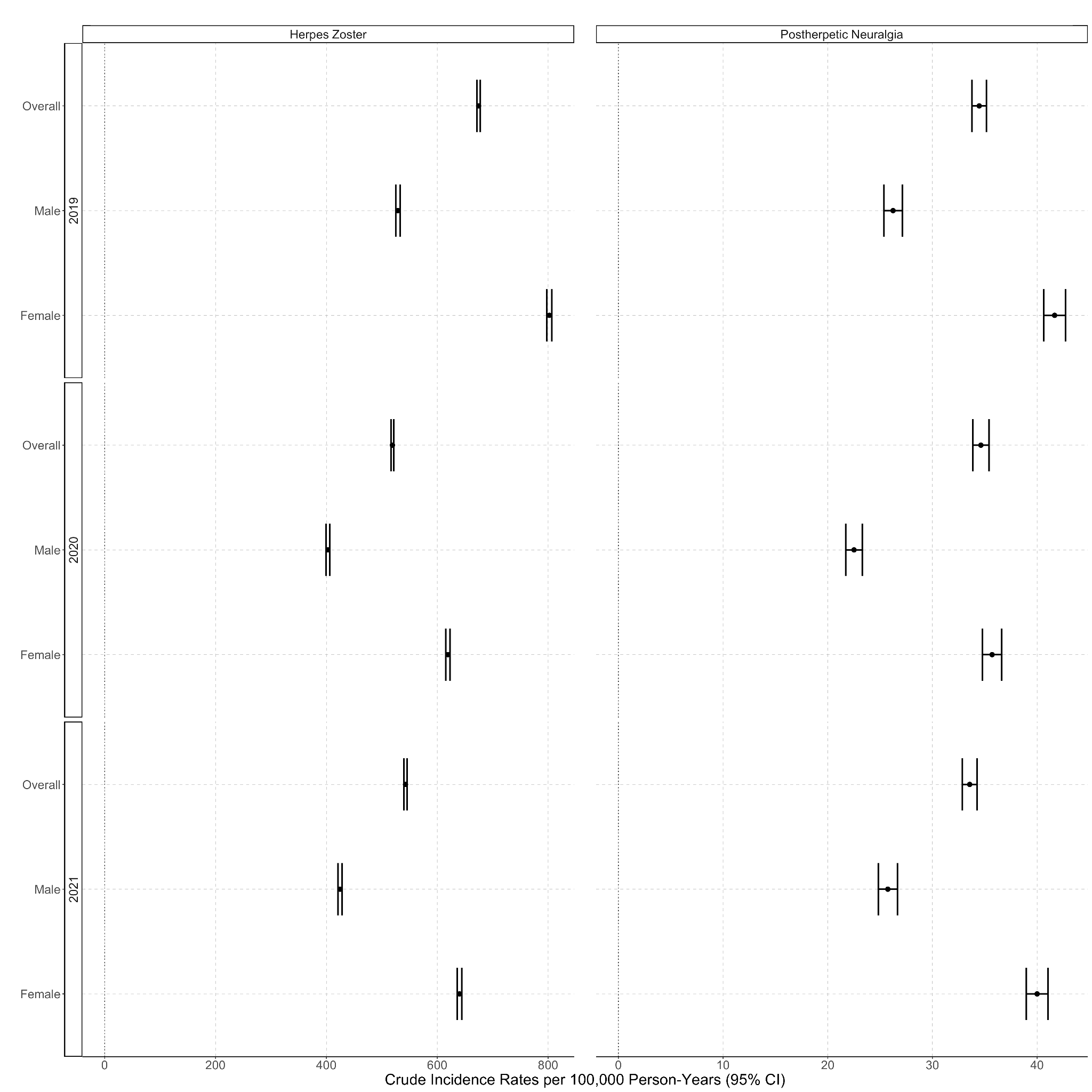


Abbreviations: CI, confidence interval.

*Note*: This figure displays the annual crude incidence rates of herpes zoster and postherpetic neuralgia from 2019 to 2021, overall and by sex.

**Supplementary Figure S4**. Crude incidence rates of herpes zoster and postherpetic neuralgia, overall and by immunocompromised status (2019, 2020, 2021).


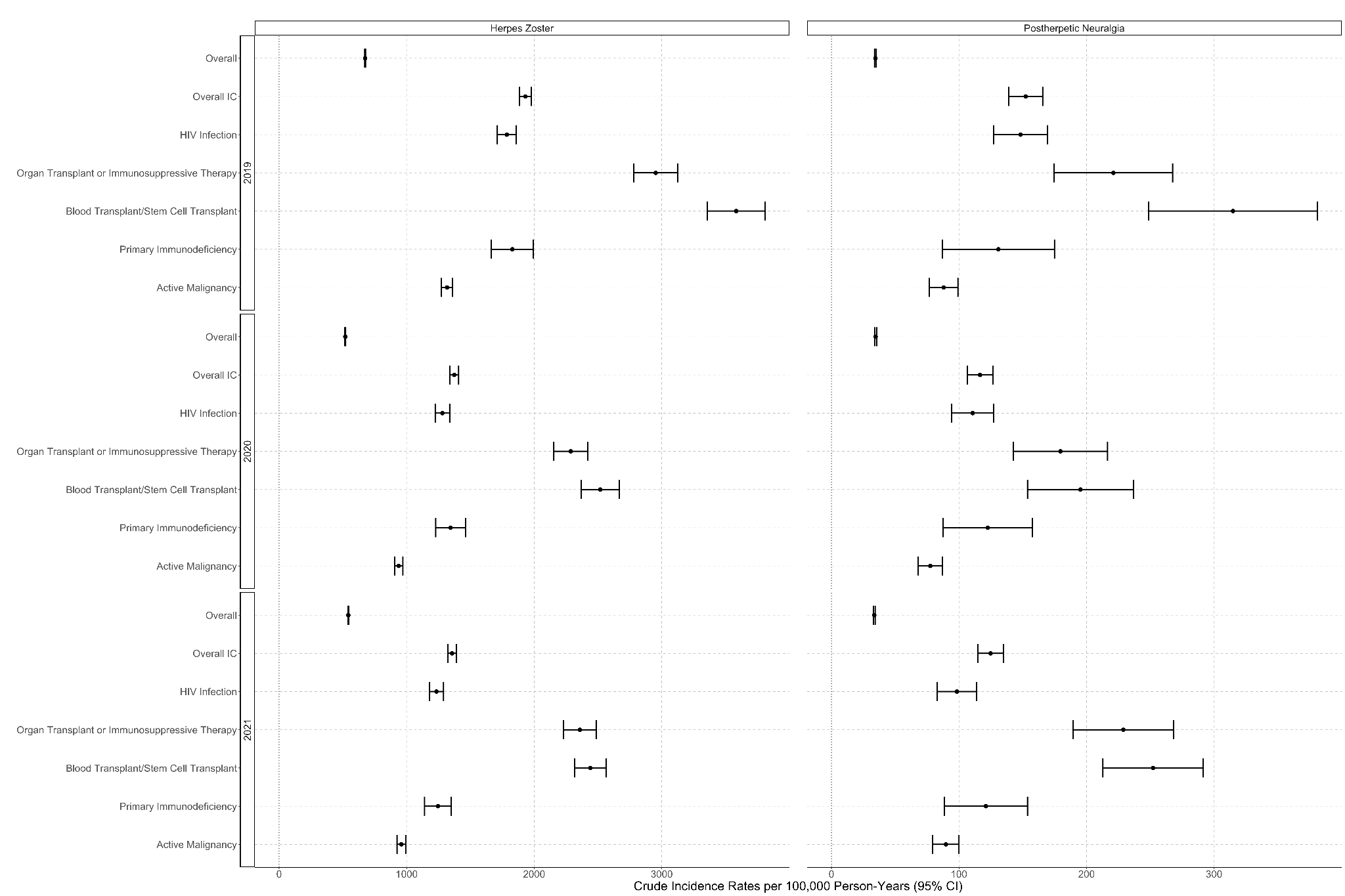


Abbreviations: CI, confidence interval; HIV, human immunodeficiency virus; IC, immunocompromised.

*Note*: This figure displays the annual crude incidence rates of herpes zoster and postherpetic neuralgia from 2019 to 2021, overall and by immunocompromised status categories.

**REFERENCES**

1. Charlson ME, Pompei P, Ales KL, MacKenzie CR. A new method of classifying prognostic comorbidity in longitudinal studies: development and validation. J Chronic Dis **1987;** 40(5):373-83.

2. Polinski JM, Weckstein AR, Batech M, et al. Durability of the single-dose Ad26. COV2. S vaccine in the prevention of COVID-19 infections and hospitalizations in the US before and during the Delta variant surge. JAMA Network Open **2022;** 5(3):e222959-e.
